# Targeting Notch to overcome radiation resistance

**DOI:** 10.18632/oncotarget.6714

**Published:** 2015-12-21

**Authors:** Sanaz Yahyanejad, Jan Theys, Marc Vooijs

**Affiliations:** ^1^ Department of Radiotherapy (MAASTRO)/GROW, School for Developmental Biology and Oncology, Maastricht University, Maastricht, The Netherlands

**Keywords:** Notch, radiotherapy, treatment resistance, Notch inhibitor, personalized treatment

## Abstract

Radiotherapy represents an important therapeutic strategy in the treatment of cancer cells. However, it often fails to eliminate all tumor cells because of the intrinsic or acquired treatment resistance, which is the most common cause of tumor recurrence. Emerging evidences suggest that the Notch signaling pathway is an important pathway mediating radiation resistance in tumor cells. Successful targeting of Notch signaling requires a thorough understanding of Notch regulation and the context-dependent interactions between Notch and other therapeutically relevant pathways. Understanding these interactions will increase our ability to design rational combination regimens that are more likely to be safe and effective. Here we summarize the role of Notch in mediating resistance to radiotherapy, the different strategies to block Notch in cancer cells and how treatment scheduling can improve tumor response. Finally, we discuss a need for reliable Notch related biomarkers in specific tumors to measure pathway activity and to allow identification of a subset of patients who are likely to benefit from Notch targeted therapies.

## INTRODUCTION

Cancer is one of the major causes of mortality worldwide. More than half of all cancer patients receive radiation therapy as part of a curative or palliative treatment often in combination with surgery or chemotherapy. While most tumors initially respond to treatment, they often acquire resistance to therapy and eventually recur. The varied clinical responses observed between and within patients are in part the result of tumor heterogeneity and both acquired and intrinsic treatment resistance often caused by deregulation of signaling pathways that control normal cell renewal in adult tissues. The Notch signaling pathway is one of these frequently altered pathways in many tumors.

The involvement of ionizing radiation in the majority of cancer treatments, the pivotal role of Notch signaling in many fundamental processes such as self-renewal and differentiation, together with the fact that Notch signaling is often deregulated in cancer, suggest that targeting the Notch pathway may be beneficial for many cancer patients. Here, we review the opportunities and challenges of targeting Notch signaling to improve treatment response to radiation therapy.

## RADIATION RESISTANCE OF CANCER CELLS

Resistance to radiation is a common phenomenon and a major obstacle in cancer therapy [1]. Intrinsic determinants of radiation resistance include pathways regulating survival and apoptosis, cell cycle status as well as DNA repair capability. Extrinsic factors including extracellular matrix molecules, cytokines, hypoxia and angiogenesis also influence radiosensitivity.

Additionally, biological heterogeneity within the tumor population leads to differential radiation response. Pre-clinical models as well as clinical observations have demonstrated substantial genotypic and phenotypic heterogeneity between (inter-tumor) and within (intra-tumor) tumors [2]. This tumor heterogeneity poses a challenge to both cancer diagnostics and therapy. First because small tumor biopsies are unlikely to capture the complete genomic landscape of a patient's tumor and thereby fail to identify (all) treatment-resistant cancer genotypes [3]. Second, while the sensitive tumor population will shrink, treatment-resistant tumor cells invariably will take over and tumors recur. Understanding factors underlying this heterogeneous treatment response will help to overcome treatment resistance.

Heterogenic treatment response may arise from influences of the microenvironment and genetic instability generating (epi)genetic changes. Malignant cell populations may alternatively (or complementary) consist of a developmentally defined hierarchy of heterogeneous phenotypes derived from a small subset of so-called cancer stem cells (CSC) [4]. Such cells have been best characterized in hematological [5], breast [6-7] and CNS malignancies [8]. Mounting evidence indicates that CSC populations in solid tumors are resistant to radiation. Mechanisms to explain this intrinsic resistance include efficient repair of DNA damage, lower numbers of DNA breaks, redistribution of cells in the cell cycle, repopulation, and reoxygenation of hypoxic tumor areas. These studies have been extensively reviewed elsewhere [9-10]. If these CSC rely on specific pathways for their survival after treatment, identification of such pathways would provide opportunities for the targeted and selective killing of treatment resistant cancer cells [11]. One of the promising candidates in this context is Notch signaling, a pathway active in many developmental as well as adult stem cell pathways and frequently altered in human cancers [12-13]

Here, we will discuss the role of Notch signaling in the radiation response of human tumors and highlight the opportunities to exploit inactivation of Notch using pharmacological inhibitors in conjunction with radiation therapy.

## NOTCH SIGNALING AND RADIATION RESISTANCE

Notch proteins are short-range cell-cell signaling receptors that have key roles during development and in adult tissue self-renewal, proliferation and differentiation. In mammals, the Notch pathway consists of 4 Notch receptors (Notch1-4) that are present in signal receiving cells and 5 ligands on adjacent signal sending cells. In the canonical pathway, Notch receptor activation *via* ligand interaction leads to a consecutive series of proteolytic cleavages finally resulting in the release of the Notch intracellular domain (NICD) that translocates to the nucleus to act as transcription regulator. The list of target genes regulated by Notch is cell type dependent and includes genes involved in cell cycle regulation [14], cellular differentiation [15] and stem cell maintenance [16].

Consistent with its fundamental role in many aspects of vertebrate development, deregulation of the Notch pathway is implicated in various developmental syndromes. In adult tissues, deregulation or mutation of NOTCH proteins is observed in many cancer types and has been shown to contribute to carcinogenesis and treatment resistance [13]. Notch inhibitors have been under pre-clinical investigation for over a decade and shown strong responses in many cancer models. Several clinical trials of Notch pathway inhibitors in patients with leukemia have been reported and several are ongoing in solid cancers [17]. Here, we focus specifically on the role of Notch in resistance to radiotherapy and the different intrinsic and extrinsic mechanisms involved.

### Intrinsic resistance

#### a) Targeting DNA repair

It has recently been demonstrated that Notch has a direct role in DNA damage response (DDR). The activity of Notch1 and ataxia-telengiectasia mutated kinase (ATM, the primary DNA sensor kinase in DDR) were shown to be inversely correlated in *C.elegans* and in human cell lines. ATM is activated specifically upon double strand (ds) DNA breaks induced by ionizing radiation. Notch1 directly binds to ATM thereby inactivating its kinase activity. Importantly, inactivation of ATM via Notch activation contribute to the survival of Notch driven human leukemia (T-ALL). Blocking Notch using a γ-secretase inhibitor (GSI) in the presence of DNA damage leads to increased radiation sensitivity in an ATM-dependent manner [18]. Activated Notch1 and pATM levels were also significantly inversely correlated in human primary breast cancer, validated by immunohistochemistry and in expression microarray datasets [18]. This result suggests that cancer cells treated with DNA-damaging agents such as radiation may undergo more robust cell death if treated with a Notch inhibitor. Another very recent and interesting observation came from a study by Deng *et al*. [19] in which they show that inactivation of homologous recombination in human Notch-driven cancer results in significant radiosensitization. This provides a basis for Notch-directed cancer therapy via blocking of homology-directed dsDNA break repair.

#### b) Targeting cancer stem cells (CSC)

There is increasing evidence supporting the role of Notch in maintenance and self-renewal of CSC in T-ALL [20-22], brain [23-24], breast [11, 25], lung [26] and colon tumors [27]. In glioma, it has been reported that blocking Notch using GSI depleted CD133+ glioma CSCs, attenuated neurosphere formation and lowered tumorigenicity [28]. In line with this, Notch inhibition selectively impaired clonogenic survival of the glioma CD133+ CSCs sub-population thereby enhancing its radiation sensitivity. The intrinsic radioresistance may be caused by alteration of DNA damage checkpoints [8] or through up-regulation of the pro-survival factors Akt and Mcl-1 in CSCs [29]. Hovinga *et al*. reported that Notch inhibition enhances the response to radiation by reducing proliferation and self-renewal of CSCs in tumor explants only when endothelial cells were present, suggesting a critical role for Notch not only in tumor cells but within the entire microenvironment [28]. Consistently, the use of Notch inhibitors in an orthotopic glioma model slowed tumor growth and prolonged survival by decreasing the number of the CD133+ CSCs [30-31].

Also in breast cancer, inhibition of Notch decreased breast CSCs (ESA+/CD44+/CD24low) activity and reduced tumor initiation *in vivo* [32]. Notch inhibition after radiotherapy prevented up-regulation of radiation-induced expression of Notch2, Notch3, Dll1, Dll3, Jag1 and was associated with a reduction in breast CSCs [33]. Radiation resistance in breast CSC has also been associated with lower levels of DNA damaging reactive oxygen species (ROS) due to increased production of free radical scavengers such as of glutathione [34]. Although a role for Notch signaling in regulating ROS in CSCs has not yet been reported, Notch inhibition in endothelial cells has been shown to increase ROS generation, proliferation, migration and adhesion, suggesting that increased ROS production upon Notch inhibition after radiotherapy could also reduce the number of breast CSCs in a non-cell autonomous manner. Also, in airway basal stem cells ROS regulates self-renewal in a Notch dependent manner [35], yet a direct relationship with the response to radiation and Notch has not been established.

In non-small cell lung cancer (NSCLC), cells with stem cell properties have also been shown to be dependent on Notch activity. These cells are more treatment resistant and tumorigenic *in vivo*, whereas GSI-treated xenografts failed to regenerate tumors upon re-implantation in suitable hosts [26]. A similar role for Notch in the maintenance and renewal of colon cancer initiating cells has been described [27]. Although in these studies, the direct role of Notch in response to radiation has not been investigated, the data suggest that Notch inhibition in these malignancies may result in improved tumor radiation sensitivity by inhibiting the viability of the cancer initiating cells.

#### c) Cross-talk with other signaling pathways

Cancers are driven by the interaction of multiple signaling pathways. Inhibiting an individual pathway will thus almost never be sufficient to cure cancer. Even in tumors that are “oncogene addicted” (referring to the dependency of some cancers on one or few genes for proliferation and survival) [36], targeting the specific genes that are critical for maintenance of the malignant phenotype will eventually result in tumor recurrence due to emergence of therapy-resistance [37]. The effect of Notch signaling on radiation response most likely also occurs through cross-talk with other signaling pathways. In glioma, Notch activity increased the radiation resistance of glioma CSCs by activating the Akt pathway. Notch blockade prior to irradiation was shown to inhibit Akt activation, an effect that was rescued by ectopic expression of the active form of Notch1 and Notch2 [29]. Likewise, in glioma spheres, Notch inhibition significantly decreased Akt and STAT3 phosphorylation and reduced survival of glioma CSCs [23]. These studies suggest that Notch can promote radiation resistance by activating Akt signaling.

In NSCLC, one of the most important overexpressed cellular targets is epidermal growth factor receptor (EGFR). Increased EGFR expression has been associated with radiation resistance [38] and combination of radiation with EGFR inhibition have yielded in relatively small but statistically significant radiosensitizing effects [39]. Others have shown that pharmacological inhibition of EGFR using erlotinib increased the stem like-cells (ALDH+) in EGFR-mutated NSCLC cell lines and that Notch transcriptional activity was increased in these cells. Strikingly, Notch inhibition eliminated the ALDH+ population, an effect attributed specifically to Notch3-dependent signaling [40]. As stem-like cells have been shown to contribute to radioresistance [41], combined EGFR/Notch targeting in lung cancer cells bearing activating mutations in EGFR could offer a very powerful approach to reduce the radiation resistant populations.

*K-RAS* is one of the most commonly mutated oncogenes in human cancer. Activated *RAS* oncogene was shown to increase radiation resistance in human cells [45]. Notch has been demonstrated to cooperate with the RAS pathway to promote carcinogenesis in various tumor types. For example, Notch1 activity was shown to be upregulated in RAS-transformed cells. Genetic or pharmacological down-regulation of Notch signaling was sufficient to abolish the RAS-induced neoplastic phenotype including proliferation and anchorage-independent growth *in vitro* and *in vivo* [46]. Taking into account the role of oncogenic *K-RAS* in radiation resistance, these data support a rational for targeting both pathways. However, caution should be taken and application of such an approach not generalized. Indeed, in a K-RAS driven NSCLC mouse model opposing tumorigenic functions of Notch1 and Notch2 were reported. In these mice, Notch1 ablation resulted in decreased levels of the Notch target gene expression and of pERK1/2, resulting in reduced tumor formation, while Notch2 ablation showed an increase in HES1 expression and resulted in increased carcinogenesis [47].

Several reports describe a direct effect of Notch signaling on the cell cycle. This may be exploited in the context of fractionated radiation, as it is well established that cells in different phases of the cell cycle exhibit different radiation sensitivity. Notch can directly induce cyclin D1 and cyclin-dependent kinase2 activity [48-49] and in breast epithelial cells Notch promotes transformation by inducing cyclin D1 [50]. *c-Myc*, an oncogene and potent driver of cell cycle entry, is a direct target of Notch and essential for cell cycle progression in T-ALL [51-52] and mouse mammary tumors [53].

#### d) Regulating EMT

There is mounting evidence showing that Notch signaling contributes to the acquisition of the EMT phenotype, for instance by up-regulating Snail and Slug, both transcriptional repressors of E-cadherin [54-55]. During EMT, epithelial cells undergo a morphological change resulting in increased motility, invasion and stemness [56] a process associated with chemo- and radiation therapy resistance [57-60]. For example, radioresistant NSCLC have been shown to share many phenotypical properties with cells that have undergone EMT [61]. Notch1 signaling was shown to enhance the EMT process in EGFR inhibitor resistant lung cancer cells [62]. In lung adenocarcinoma, a population of metastasis-prone cells with significantly enriched expression of Notch receptors and ligands that drive EMT were identified [63]. While the majority of the metastatic lung cancer cells were shown to be radioresistant [64], Notch targeting suggests a role in increasing radiation sensitivity by inhibiting genes involved in the EMT process.

In NSCLC, c-MET amplification is shown to direct invasion and metastasis [65]. Others have shown that co-expression of c-MET and Notch1 induces EMT in NSCLC patients and promotes invasion [66], likely due to their interaction by cross-talk. Given the role of Notch and c-MET expression in poor radiation response [67] as well as direct interaction between c-MET and Notch [68], Notch blocking in combination with c-MET targeted therapy could be critical to inhibit the aggressive behavior of NSCLCs and increase the radiation sensitivity.

The association between EMT and radioresistance and the prominent role of Notch signaling as driving force in the EMT process, suggest that Notch inhibition will result in radiosensitization of tumors that underwent EMT.

## CROSS-TALK WITH MICRORNAS

Increasing evidence implicates microRNAs (miRNA) in the regulation of drug and radiation resistance [69-73]. The most extensively studied miRNA in the context of Notch signaling and cancer is the tumor suppressive miR-34. In glioma, miR-34 was shown to inhibit tumor growth *in vivo* by down-regulating Notch1 and Notch2 expression [73]. Similarly, in colorectal cancer, high miR-34a levels inhibited colon CSCs self-renewal *in vitro* as well as xenograft tumor formation by suppressing Notch signaling whereas low miR-34a levels up-regulated Notch and promoted a CSC phenotype [74]. Also in NSCLC the expression of miR-34 was reported to be low [75] and its ectopic expression enhanced the radiation sensitivity of lung cancer cells [76]. Kang *et al*. showed that this effect is Notch1-dependent and demonstrated that miR-34 induced Notch1 downregulation thereby promoting apoptosis resulting in a radiosensitizing effect [77]. Similarly, in P53-deficient gastric and pancreatic cancer cells, restoration of miR-34 reduced the expression of Notch pathway members and was associated with reduced *in vivo* tumor formation as well as increased treatment sensitization [78-79]. Overall, these studies provide insight on the role of miR-34 in radiation resistance, partly mediated via regulation of Notch.

### Extrinsic resistance

#### a) Angiogenesis

Notch signaling is important in pro-angiogenic role in tumor vasculature. Endothelial cells (ECs) express several Notch receptors (Notch1, 4) and ligands (Delta-like 1, 4 and Jagged1). VEGF acts as a proliferative driver of angiogenesis, while Dll4/Notch signaling helps to control vessel sprouting and branching [80-81]. Tumor vasculature is abnormal, and the endothelial cells of tumor blood vessels are different from those of normal vasculature [82]. Consequently, increased tumor angiogenesis, as indicated by increased microvessel density or by increased VEGF expression, does not necessarily correlate with increased blood flow and oxygen availability. This situation, together with the existence of heterogeneous hypoxic regions within tumors results into reduced response to radiation therapy. Based on the crucial role of Dll4/Notch signaling in the vascular sprouting and tumor angiogenesis, pharmacological targeting of the Dll4/Notch has been shown to be effective as a novel anti-angiogenic therapy by blocking non-productive vessel growth and tumor collapse [83-85]. Adding chemotherapeutic agents to the Dll4 cocktail inhibited the induction of anti-apoptotic genes and resulted in further additive anti-tumor activity by decreasing the CSC population. Also when combined with ionizing radiation, Dll4 blockade impaired the tumor growth by promoting non-functional tumor angiogenesis [86]. As it is the case for all anti-angiogenic treatment, the temporal relationship with radiation is crucial [87] and therefore the optimal order and timing for administration of the radiation/anti-Dll4 combination in the context of vascular normalization requires careful attention (see below).

#### b) Hypoxia

Hypoxia is a common feature of human tumors and is associated with increased malignancy and resistance to chemo- and radiotherapy [88]. Hypoxic cells are 2-3 fold less sensitive to the effects of radiation because they lack the oxygen radicals that contribute to irreversible DNA damage [89]. Pre-treatment oxygenation of tumors is prognostic and predictive for radiotherapy response in head and neck squamous carcinoma and many other tumors [90-91]. Previously, we have shown that NSCLC xenografts expressing a constitutively active Notch1 were more resistant to single dose radiation therapy. Tumors with high constitutive Notch activity proliferated faster and consistently had a higher hypoxic fraction [92]. This was accompanied by increased vessel density while the total number of perfused vessels remained similar to control tumor cells pointing towards an increase in non-functional vasculature (unpublished data). These data suggest that Notch signaling may increase the survival of hypoxic cells and thereby influence the response to radiotherapy.

The link between hypoxia and Notch signaling has been described in various studies. Notch has been reported to activate the hypoxia response pathway through HES1-induced STAT3 phosphorylation resulting in transcriptional up-regulation of HIF1-α and its target genes [93]. Interestingly, hypoxia in turn also elevates Notch activity [94-95] both via HIF1-α dependent [94] and HIF1-α independent pathways [96]. Especially the latter is intriguing, as it was shown that hypoxia-induced Notch signaling contributed to increased proliferation, maintenance of stem cell properties and suppression of senescence via a metabolic shift to glycolysis [96]. Glycolysis would not only provide a growth advantage for the fast proliferating cells but is also involved in cellular immortalization via reduction of intrinsic ROS [97-99]. As such, the metabolic effects of Notch on glycolysis may be indirectly responsible for increasing survival and radiation resistance by suppressing ROS.

Although many of these oncogenic pathways are found to cross-talk with Notch at some level, these interactions are cell type and context dependent. Thus, the direct effect of Notch blockade on cancer cells may vary.

## STRATEGIES TO TARGET NOTCH AND ITS POTENTIAL RISKS

In most cases, deregulation of Notch has oncogenic effects. The first evidence for the involvement of Notch in cancer was the detection of a rearrangement between the intracellular part of Notch1 (NICD1) and the T-cell receptor beta (TRB) leading to high-level expression of truncated and constitutively active Notch1 in T-ALL [100-101]. Mutations and chromosomal rearrangements have also been reported in splenic marginal zone B-cell lymphoma [102-103] and in triple-negative breast cancer cells [104]. Notch has also been shown to act as oncogene in lung [26, 92], colon [105], melanoma [106], pancreatic [107], glioma [31, 108], head and neck [109] and many other cancer types.

In other cases, Notch functions as a tumor suppressor as shown in skin tumors [110-111], bladder cancer [112], squamous cell lung carcinoma [113], squamous cell carcinoma in cutaneous and head-and-neck tumors [114-117] and potentially also in small cell lung carcinoma (SCLC), which is a neuroendocrine subtype of lung cancer [118].

This indicates that the outcome for aberrant Notch activity is highly context-dependent. Notch inhibitors are being investigated in clinical studies to treat these malignancies where Notch acts as an oncogene whereas methodologies activating the Notch pathway may have therapeutic potential in those cancers where Notch suppresses tumor growth, although any experimental evidence for the latter is still lacking.

**Figure 1:**
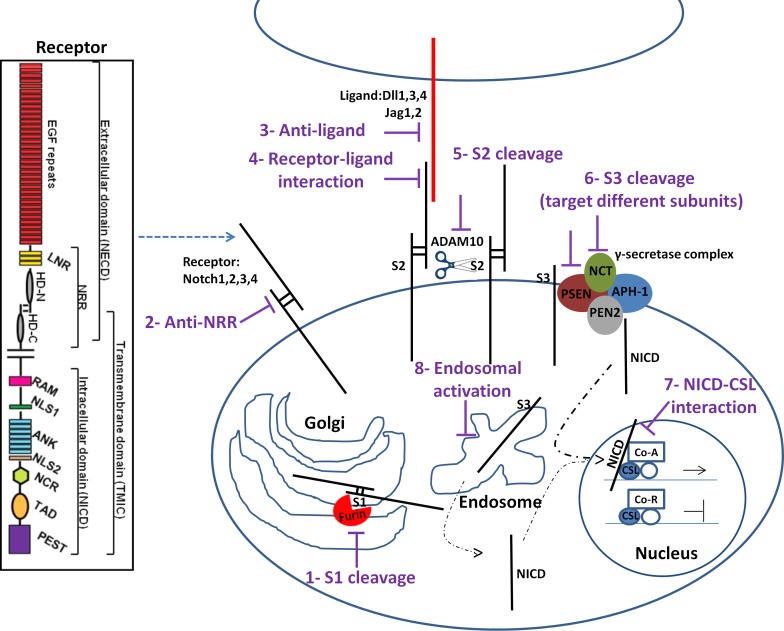
Notch signaling pathway and potential drug intervention sites (see text for details) 1) Furin cleavage at S1 site can be inhibited. 2) Notch antibodies targeting Notch receptors 3) or ligands would target individual receptor pathway 4) Targeting the interaction of Notch receptor with ligand after receptor maturation abrogates the pathway activity 5) Cleavage by a disintegrin and metalloproteinase Adam10 at S2 site and 6) γ-secretase complex at S3 site can be inhibited to limit Notch signaling. 7) Interfering with NICD/CSL interaction using small peptides disrupts the canonical Notch pathway signaling. 8) Inhibition of endosomal Notch trafficking could potentially reduce Notch signaling activity regardless of ligand activity. The Notch receptor is comprised of a Notch extracellular domain (NECD) and Notch intracellular domain (NICD). EGFR: epidermal growth factor repeats; HD: heterodimerization domain; NRR: negative regulatory region; LNR: cysteine-rich LNR repeats; RAM: RAM domain; NLS: nuclear localization signals; ANK: ankyrin repeat domain; NCR: cysteine response region; TAD: transactivation domain; PEST: region rich in proline (P), glutamine (E), serine (S) and threonine (T) residues.

We will first provide an overview of the different possibilities to inhibit the Notch pathway (Figure 1) and discuss how these may interact with radiation in a subsequent section. In canonical Notch signaling, Notch receptors are subjected to a series of sequential proteolytic cleavages. The site1 (S1) cleavage is controlled by a Furin convertase and is responsible for the receptor maturation. Therefore, it is possible to interfere with Notch maturation in Golgi by using Furin inhibitors [119-120]. After receptor maturation, the receptor is transported to the cell's surface, a process that can be blocked by using an inhibitor of calcium transporter Atp2a3/SERCA [121]. Following surface expression, receptor activation is mediated by binding to ligand on adjacent cells. Notch-ligand interactions can be blocked using soluble versions of the receptor that function as decoy [122-123] or by blocking the ligand-induced conformational changes in the Notch receptor [124-126]. Upon receptor-ligand interaction, mammalian Notch receptors are cleaved by the disintegrin metalloprotease ADAM10 at site 2 (S2) [127-128] leading to shedding of the large Notch ectodomain (NECD). This cleavage is followed by a S3-cleavage caused by a γ-secretase complex and results in the release of the cytoplasmic NICD, which subsequently translocates to the nucleus where it binds to the DNA binding protein CSL (CBF1/Suppressor of Hairless/Lag-1; also known as Rbp-j) and the co-activator Mastermind-like (MAML) to induce expression of target genes [128-129]. The S2 cleavage can be inhibited by blocking ADAM proteases [128, 130-132] and S3 cleavage by γ-secretase inhibitors (GSI).

A detailed overview of ways to intervene with Notch signaling in various disease has been described elsewhere [17]. Here, we focus on different strategies to target Notch specifically in cancer.

*1- GSIs*: GSIs are pan-Notch inhibitors used in both pre-clinical and clinical settings. They target γ-secretase cleavage of NOTCH by presenilin, a rate-limiting step in the Notch activation cascade. Use of GSI is however hampered by the dose-limiting toxicity in the gut as Notch inhibitors promote goblet cell metaplasia leading to severe diarrhea in animals and humans [133-135]. Intermittent dosing schedules [136-138], glucocorticoid administration or anti-estrogen therapy [139] have been shown to mitigate the adverse effects while maintaining GSI's anti-tumor efficacy [140-141]. Obviously, most drugs used in oncology, including targeted agents and immunotherapeutics have significant acute and chronic toxicities, and in every situation the ratio between risks and benefits must be carefully weighted. Cyclical (intermittent) dosing, dose adjustments and patient stratification are therefore important to minimize the toxicities of chemo- and radiation therapy [139, 142-146].

While GSI's are potent inhibitors of the Notch signaling pathway, they are not designed to be receptor-specific and they target all four Notch isoforms that within the same cell type can have either tumor promoting or tumor suppressive roles. Thus, there is a need for receptor specific antagonists.

*2- Notch receptor specific targeting:* Monoclonal antibodies have been developed that target the negative regulatory region (NRR) of Notch1, Notch2 [125] and Notch2/3 [126, 147] and act by keeping the receptor in an unresponsive “closed” confirmation or by blocking receptor-ligand interactions through hindering EGF repeats required for binding [148]. These antibodies are able to target cancers by inhibiting simultaneously cancer cell growth and by disrupting tumor angiogenesis that depends on DLL4/Notch1 signaling.

*3- Notch ligand targeting*: To interfere with tumor angiogenesis, Notch ligands are important targets. Along this line, Dll4 blocking antibodies have been used to suppress tumor vascularization and tumor growth [83, 149]. Data from a Phase1 clinical trial showed that Demsizumab (anti-Dll4) suppressed tumor vascularization, was well tolerated and resulted in reduced tumor size [150-151]. In animals, the long-term use of these antibodies, however caused marked histopathological changes in liver endothelial cells and induced vascular tumors [152]. While Dll4 mainly has a function in the vasculature, Jagged1 is important in immunosuppressive T regulatory cells and promotes the maintenance or expansion of hematopoietic precursor cells [153] as well as tumor/stem cells [154-155]. Targeting Jagged1 in stroma and tumor cells can thus result in synergistic effects as demonstrated in ovarian cancer [54, 156].

Alternatively, activating Notch signaling could be a way to inhibit angiogenesis. While overexpression of Dll4 was shown to promote tumor growth, overexpression of a soluble DSL domain of Dll1 resulted in reduced tumor growth by attenuating vascularization [157]. Therefore, it is important to further explore the differential activities of the Notch ligands on both stroma and tumor cells.

Recent work has shown that the Notch pathway can also be used non-canonically [158] and that Notch proteins can become activated in a DSL-independent manner as shown in various cancers including melanoma [159] and T-ALL [160]. Breast cancer stem cell expansion has also been shown to be dependent on a ligand-independent Notch activation mechanism [161]. Taking into account the radioresistant phenotype of breast CSC, targeting Notch ligands in such case may be suboptimal, and other strategies to target Notch cleavage or downstream events may be more effective.

**Table 1:** Possible sites to intervene in Notch pathway

Intervening Notch at various sites	Mechanism of action	Pre-clinical studies	Clinical studies
S1 cleavage	- Inhibition of Furin and block receptor maturation	- Not reported (experimental studies available)	- Not Reported
Receptor -Anti Notch1, Notch2, Notch3 -Anti-Notch4	- Unresponsive receptor to ligand binding by targeting NRR in case of Notch1,2 and 3 - Blocking receptor–ligand interactions by hindering EGF repeats required for binding	- Anti-Notch1, Notch2 [125, 202] - Anti-Notch3 [147] - Anti-Notch4 [203]	- Anti-Notch2, Notch3 [138] - Anti-Notch1 [204]
Ligand -Anti-Dll4 -Anti-Jagged1	- Non-functional vasculature - Abrogation of angiogenesis, targeting CSCs, targeting EMT and inhibiting the immunosuppressive T- regulatory cells	- [83] - [156, 205]	- [151] - Not reported
Receptor-Ligand Interaction -Notch1 receptor decoy -Dll1 and Jag1 ligand decoy	- Ligand dependent Notch antagonist	- [122]	- Not reported
S2 cleavage (Adam metalloproteases)	- Targeting both Adam metalloprotease 10/17 and block ectodomain shedding	- [131]	- [206-207]
S3 cleavage (γ-secretase complex)	- Inhibition of different subunits of γ-secretase complex and block NICD release	- [208]	- [209]
NICD-CSL interaction	- Suppressing transcriptional activation by preventing binding of MAML1 to the ICN–CSL complex	- [174, 210]	- Not reported
Endosomal activation	- Disrupting γ-secretase cleavage in acidic endosome - Inhibition of V-ATPase	- [211] - [173]	- Not Reported

4- *Alternatives*: Cleavage of Notch proteins by ADAM metalloproteases is a rate-limiting step preceding γ-secretase cleavage. Specifically, metalloproteases ADAM 10 and 17 have been implicated in ligand dependent and independent signaling, respectively [127, 162-163]. ADAM metalloproteases bind many signaling molecules and receptors, including TNFα and EGF receptors and thus unless specifically targeted to tumors are likely to yield dose-limiting toxicities in normal tissues. Moreover, we reported Notch cleavage and transcriptional activity of oncogenic Notch1 signaling in cells treated with both broad-spectrum metalloprotease inhibitors as well as specific ADAM17/10 hydroxamate inhibitors [128], suggesting the involvement of unknown proteases engaged in the activation of oncogenic Notch1. While this hypothesis requires further validation, it may open the possibility of targeting disease-specific Notch proteases while leaving normal Notch signaling intact.

The main components of the γ-secretase complex are presenilin, APH-1, Pen-2, and Nicastrin. Currently used GSIs inhibit the catalytic activity of presenilin and lead to off-target effects on the wide range of γ-secretase complex substrates. [164]. Targeting Nicastrin as the key component of the γ-secretase complex using neutralizing antibodies reduced proliferation of cancer cells [165] and resulted in anti-tumor and anti-metastatic effects in a model of triple negative breast cancer [166].

γ-secretase cleavage and activity not only occurs at the cell surface but also in the acidic environment of the endosomes and lysosomes [167-168] where the activity of vacuolar ATPase (V-ATPase) regulates acidification of endocytic compartments necessary for Notch signaling activation [23, 169]. Endocytic trafficking is also essential for Notch ligand internalization and promoting Notch activation [170-171]. Pharmacologic inhibition of V-ATPase decreases Notch signaling activity [172] and pretreatment with V-ATPase inhibitors can sensitize solid human tumors to chemotherapy drugs and might also a be a good strategy for radiosensitization [173].

Finally, hydrocarbon-stapled peptides that mimic a dominant negative fragment of Notch-CSL-mastermind-like (dnMAML) and that prevent binding of full-length MAML to NICD/CSL have been developed. Unlike GSIs, these peptides have shown a reduced gastrointestinal toxicity in treated animals [174].

### Treatment scheduling and personalized treatment

While numerous clinical trials using various Notch inhibitors were ongoing, several of these trials have stopped due to dose-limiting toxicity and lack of efficacy. Combining Notch inhibition with (chemo) radiation can only be successful if these hurdles can be overcome. Appropriate treatment scheduling and patient selection will be key to achieve this goal.

#### a) Treatment scheduling

One of the most important aspects that have been understudied is the scheduling for Notch inhibitors in conjunction with other treatments. For example, it has been reported that Notch inhibition caused hypersprouting of non-functional vasculature resulting in decreased tumor growth [175-176]. Impaired angiogenesis has also in other studies been shown to reduce tumor growth, yet at the same time, these tumors became strongly hypoxic [177]. Thus, administration of a Notch inhibitor in patients before radiotherapy may induce hypoxia and contribute to a more malignant phenotype and radio- and chemotherapy resistance [88].

It has also been shown that irradiation can induce Notch expression and activity and promote stem cell like characteristics [7, 33, 178-180]. Therefore, it may be critically important to continue Notch inhibition after radiotherapy. Significantly enhanced radiation-mediated tumor cytotoxicity has indeed been demonstrated upon treatment with GSI following irradiation in lung xenografts [178]. A similar study in glioma determined that Notch inhibition before temozolomide administration diminished the efficacy of chemotherapy while Notch inhibition after chemotherapy strongly inhibited tumor formation [181]. The reason for this could likely be due to the induced Notch activity after chemotherapy treatment [47]. More data are clearly needed to determine the most appropriate treatment schedule and such results will be invaluable for translation into the clinic with the aim to improve outcome. In colorectal cancer patients, sequence of the drug treatment was shown to be more important and effective than the drug exposure itself likely due to enhancing the subsequent treatment [182].

It will also be of utmost importance to determine the interaction between Notch inhibition, chemotherapy and fractionated radiation. Recovery from radiation injury and tumor cell repopulation between fractions reduces tumor control, while reoxygenation of hypoxic cells and redistribution of cells into a more radiosensitive phase increases tumor control [183-184]. In addition, the effect on the therapeutic ratio when Notch inhibition will be combined with alternative fractionation schedules such as accelerated (decreasing the overall treatment time) or hypofractionated (lower number of fractions with a higher dose per fraction) treatment needs careful attention as such alternative fractionation schemes have been shown to improve tumor control [185-186].

Alternatively, increased radiotherapy effectiveness can potentially also be achieved by properly scheduling in combination with angiostatic drugs such as anti-Dll4 antibodies through “vascular normalization”. This concept proposes that in order to have an effect when using anti-angiogenesis drugs, an equilibrium between pro- and anti-angiogenic factors in the tumor microenvironment rather than complete angiogenesis inhibition is needed. Dysfunctional vasculatures become then more normal, hence tumor oxygenation and perfusion will be improved thereby increasing the efficacy of administered drugs and/or radiation [187]. Defining the optimal time point during radiotherapy at which anti-angiogenesis drugs such as Notch inhibitors should be administered will be crucial [188].

#### b) Genetic profile of cancer types and signatures

The different classes of gene expression profiles, reflecting the consequences of different sets of oncogenic mutations, correlate with different prognoses and different responses to therapy. Therefore, cancer cells vary widely in their response to radiation therapy [189] as well as Notch targeted therapies reflecting their particular genetic profile. For example, estrogen receptor negative (ERα^−^) breast cancer cells have higher Notch activity and respond better to Notch inhibition. In ERα^+^ cells when estrogen is deprived or upon anti-estrogen-treatment, breast cancer stem cells are selectively enriched and Notch-4 activity increased [190-191]. Combination of a Notch inhibitor with an anti-estrogen could therefore be a promising therapeutic strategy in ERα^+^ breast cancer cells. In both ERα^−^ and ERα^+^ tumors radiosensitivity is expected to increase, as Notch inhibition will specifically target the more radioresistant stem cell compartment. Yet, there are currently no valid predictive factors that reliably identify patients who would greatly benefit from radiation treatment. In triple-negative breast cancer (TNBC) patients, approximately 19.5% carry *BRCA* mutations [192]. These mutation carriers are defective in DNA repair; therefore, it would be expected that these tumors might exhibit sensitivity rather than insensitivity to radiation therapy. One possible explanation for this response is that these tumors might possess compensatory DNA repair mechanisms that are more effective at dealing with radiation-induced DNA damage. In this regard, identification of a marker in TNBC cells would be invaluable in identifying potential radiosensitizing agents. Gene expression profiling analysis performed on these tumors revealed that oncogenic PEST domain mutations in Notch1, 2 and 3 receptors occur in ∼13% of TNBC cells conferring GSI sensitivity [104] and provides a strong rationale for a Notch-driven personalized medicine strategy. Notch4, but not Notch1-3 was shown to contribute to the induction of proliferation, tumorigenesis and invasiveness in TNBC cells and its inhibition was shown to suppress tumorigenicity and tumor volume [193-194]. Furthermore, mutations of Notch receptors resulting in an active Notch pathway are frequent in TNBC conferring GSI sensitivity [104]. Notch targeting can thus be a potential therapeutic target for the radiosensitization of TNBC cells.

In skin squamous cell carcinomas (SCCs), EGFR signaling plays a significant role in suppressing differentiation through negative regulation of Notch1 gene expression and activity [195]. Especially for large skin SCC and at sites where surgery is not an option, radiation is often used as first-line treatment. Notch blockade counteracts the differentiation-inducing effects of EGFR inhibitors, while at the same time, synergizes with these compounds in induction of apoptosis. This indicates an attractive combination therapy that may enhance the potency of EGFR inhibitory agent. This study provides a mechanistic explanation for the Notch loss-of-function mutations found in squamous skin carcinomas [113]. Squamous tumors without such mutations may thus be sensitive to Notch inhibitors, and treatment efficacy enhanced especially for indications involving radiotherapy.

In NSCLC patients, Dll4/Notch1 signaling was reported to negatively influence NSCLC growth via PTEN up-regulation [97]. This indicates that the therapeutic application of a Notch inhibitor could be adversely affected in different categories of lung cancer [196]. Notch inhibition could be specifically beneficial in lung cancers with inactive PTEN [197]. In contrast, in glioma, loss of PTEN has been reported as a critical event that leads to Notch inhibitor resistance by transferring the “oncogene addiction” from the Notch to the PI3K/AKT pathway [198], supporting the regulatory link between Notch and the PTEN/PI3K/AKT pathway. Therefore, attenuation of cell growth using a combination of Notch inhibition and PI3K inhibitors in PTEN mutant glioma CSCs may lead to increased treatment efficacy [199]. As glioma stem cells promote radioresistance by preferential activation of the DNA damage response [8] and Notch has been shown to enhance radiation resitance in glioma [29], the combination of Notch inhibitors with radiation can be expected to yield beneficial outcomes in these patients.

These data and similar other data arising from genomic, transcriptional and proteomic analysis in glioma [31] or breast cancer [200] exemplify how understanding the molecular signatures that could predict the therapeutic response allow identification of a subset of patients who are likely to benefit from the Notch inhibition/radiotherapy combination therapies.

Patient selection could also be performed based on determination of activated (i.e. cleaved) Notch proteins levels or target genes as shown in TNBC [201], indicating their potential as prognostic biomarker to identify TNBC patients who are most likely to respond to anti-Notch based therapeutics. Likewise, adenoid cystic carcinoma (ACC) tumor xenografts with activating Notch1 mutations responded to Notch inhibition, whereas the tumors without Notch1 mutation and low levels of NICD1 were resistant [201]. Therefore, establishing an association between the drug responses and molecular subclasses of the specific cancer type may help to identify potential cohorts of patients for targeted therapy and to be treated in combination with radiotherapy.

Taken together, while Notch deregulation is frequent in cancers, the failure of clinical trials using Notch inhibitors may be explained by our incomplete understanding of the unique and redundant functions of the Notch receptors and our inability to select the correct patients and lack of knowledge on the correct timing of intervention. However, it appears that Notch signaling plays a key role in tumor initiation, progression and treatment response and that combining Notch therapeutics with radiotherapy may lead to synergistic improvements. More basic and translational research is needed to address these issues prior to conducting clinical trials. Only then can we expect to see therapeutic profit from Notch inhibitors on cancer response.
